# Bacterial microbiota similarity between predators and prey in a blue tit trophic network

**DOI:** 10.1038/s41396-020-00836-3

**Published:** 2021-02-12

**Authors:** Hélène Dion-Phénix, Anne Charmantier, Christophe de Franceschi, Geneviève Bourret, Steven W. Kembel, Denis Réale

**Affiliations:** 1 Département des sciences biologiques, Université du Québec à Montréal, Montréal, QC Canada; 2 CEFE, Univ Montpellier, CNRS, EPHE, IRD, Univ Paul Valéry Montpellier 3, Montpellier, France

**Keywords:** Food webs, Microbial ecology

## Abstract

Trophic networks are composed of many organisms hosting microbiota that interact with their hosts and with each other. Yet, our knowledge of the factors driving variation in microbiota and their interactions in wild communities is limited. To investigate the relation among host microbiota across a trophic network, we studied the bacterial microbiota of two species of primary producers (downy and holm oaks), a primary consumer (caterpillars), and a secondary consumer (blue tits) at nine sites in Corsica. To quantify bacterial microbiota, we amplified 16S rRNA gene sequences in blue tit feces, caterpillars, and leaf samples. Our results showed that hosts from adjacent trophic levels had a more similar bacterial microbiota than hosts separated by two trophic levels. Our results also revealed a difference between bacterial microbiota present on the two oak species, and among leaves from different sites. The main drivers of bacterial microbiota variation within each trophic level differed across spatial scales, and sharing the same tree or nest box increased similarity in bacterial microbiota for caterpillars and blue tits. This study quantifies host microbiota interactions across a three-level trophic network and illustrates how the factors shaping bacterial microbiota composition vary among different hosts.

## Introduction

Host-associated microbes play key roles in host digestion [1], immune response [2, 3], growth [4, 5], pathogen defense [6], and behavior [7]. The vast majority of studies on animal-associated microbiota focus on humans, livestock, and lab-reared animals [8]. However, it is known that captivity modifies host microbiota and that comparisons between captive host and natural host microbiota are difficult [9]. There is a need for microbiota studies in the wild because of the direct link between microbiota and host evolution and fitness [8]. Among studies in wild animal populations and communities, the gut microbiota is often investigated and to a lesser extent, skin microbiota and oral microbiota. Host species seems to be the most important factor determining gut microbiota composition [10, 11]. Studies have also shown the important role of multiple environmental factors at different scales [12, 13] and host diet in shaping microbiota [14, 15]. A host diet can affect its microbiota due to its nutritional or chemical composition, but also through the ingestion of the bacteria that it contains. Few studies, however, have compared the microbiota of a predator and its prey to evaluate the potential for transmission between trophic levels. Some bacteria may be transferred from cabbage root fly to its parasitoids [16] while some insects acquire a large part of their microbiota from the plants they feed on [17, 18]. To our knowledge, no study has investigated the microbiota of multiple hosts along a trophic network composed of primary producers, primary consumers, and secondary consumers, which may help us evaluate the potential for bacterial transfer and dilution along that network. Evaluating the potential for transfer of microbes between species is important for several reasons. For example, the microbiota affects many features of its hosts (see above), and transmission along a network may thus help us explain differences in the ecology of communities or populations of the same species. It may also be important to examine the probability of transfer of a microbe along several levels of a trophic network to evaluate to what extent the structure of that network is permeable to the transmission of diseases [19].

In this study, we analyzed the bacterial microbiota of three types of hosts along a simple trophic network: leaves of two tree species, folivorous caterpillars on these trees, and blue tits preying upon these caterpillars and using them to feed their nestlings.

Trees host microbiota in and on their roots, stems, and leaves [20]. Leaves are densely populated by bacteria [21] although ultraviolet radiation exposure, temperature fluctuations, and low water and nutrient availability make leaves a challenging environment for bacteria [22]. Leaf microbiota composition varies strongly among tree species [23–26] and to a lesser extent along spatial and environmental gradients [24, 25].

Lepidopteran caterpillars host microbiota on their skin, in their body, and in their guts. The caterpillar gut has a low pH, simple morphology, and fast transit time [27] and bacterial growth is minimal or absent in caterpillar gut [28–30]. Thus, it has been suggested that caterpillar gut microbiota mainly come from their diet [31, 32]. Furthermore, it can vary strongly between individuals [18, 33, 34]. Caterpillar gut microbiota also vary among host species and locations [32, 34]. Hannula et al. [18] found that caterpillars feeding on plants that are still in the soil have a more complex and diverse microbiota than caterpillars feeding on detached leaves that have grown in the same soil. Thus, beyond the diet caterpillars may acquire bacteria directly from the environment. Taken together, these results suggest that caterpillar gut microbiota may not contain many host-associated microbes and may be more subject to variation due to external factors than other host types [33].

In contrast to the relatively well-studied mammal microbiota, there are fewer studies focusing on the factors structuring the avian gut microbiota. The environment around the nest [13, 35] and in the nest [36, 37] can affect the avian microbiota. Several studies have highlighted the importance of diet in shaping the avian microbiota, especially the gut microbiota [11, 38–40]. Long-term habits and nutritional contents are more important for determining microbial composition than short-term variation in diet [15]. Some short-term variation, however, has also been observed [41]. Birds can also transfer bacteria to each other through direct contact [19, 42]. Avian microbiota are more influenced by extrinsic factors such as environment and diet than by intrinsic factors such as host genetics and evolutionary history [11, 13]. However, these extrinsic factors could be considered part of the extended phenotype of the host [43, 44]. Nest materials, for example, are chosen by the parents and nest material preferences can be partly genetic [45].

In a trophic network framework, we studied the bacterial microbiota of (1) leaves from two oak species, (2) leaf-feeding caterpillars, and (3) blue tit feces, in nine study sites in Corsica. During their breeding period, blue tits feed their young and themselves mainly on leaf-eating lepidopteran caterpillars, specifically the abundant green oak tortrix (*Tordix viridana*) [46]. The important and well-known relation between oaks, caterpillars and titmice [47, 48] makes this study system ideal for the study of microbiota along a trophic network.

We investigated the spatial structure of the bacterial microbiota in this trophic network at the forest stand, the site, and the nest box scales. The similarity of the microbiota between two host types could be driven by bacterial transfer through diet [18] and through the shared environment. Due to the potential for microbiota transfer through diet, we predicted that bacterial microbiota would be more similar between predators and prey of adjacent trophic levels (i.e., leaves-caterpillars and caterpillars-blue tits) than bacterial microbiota from hosts separated by two trophic levels (i.e., leaves-blue tits).

Our spatially structured study design gave us the opportunity to compare environmental effects at different spatial scales for the bacterial microbiota of each host type. Because tree species are the main driver affecting leaf microbiota [23–26], we expected a difference between bacterial microbiota found on deciduous downy oaks and on evergreen holm oaks. Also, because of the shared environment and the diet transfer along the trophic networks, we expected differences in caterpillar and blue tit bacterial microbiota between deciduous and evergreen habitats. We thought that environmental factors associated with site and nest box location might also affect microbiota. Thus, we expected that the bacterial microbiota composition of individuals of each host type would be more similar within a site or a nest box than with the individuals sampled in other sites or nest boxes.

## Materials and methods

### Study species and sites

This study was conducted at nine study sites (between 22,000 m^2^ and 500,000 m^2^) situated in two valleys (Regino and Fango) near Calvi, island of Corsica, France where artificial nest boxes were installed for long-term monitoring of Corsican blue tit (*Cyanistes caeruleus oligastra*) reproduction. Blue tits are small (9–13 g) cavity-nesting, socially monogamous passerines commonly found in wooded habitats of the western Palearctic [49]. Three sites (Pirio, Tuarelli, and Mont-Estremo) in the Fango valley (42°34′N, 08°44′E; 200 m elevation; 205 nest boxes) and three sites (Grassa, Arinelle, and Filagna) in the Regino valley (42°35′N, 08°57′E; 100 m elevation; 75 nest boxes) are dominated by evergreen holm oak (*Quercus ilex*). Three sites (Muro, Avapessa, and Feliceto) in the Regino valley (42°32′N, 08°55′E; 350 m elevation; 110 nest boxes) are dominated by deciduous downy oak (*Quercus pubescens*). The Regino valley and the Fango valley are separated by 24.2 km. The leafing process of deciduous oaks occurs in mid-April and about three weeks later for evergreen oaks [47]. Spring peak abundance of leaf-eating caterpillars, specifically the abundant *Tortix viridana*, is higher and earlier in deciduous habitats, and occurs about 2 weeks after bud burst, while it occurs 3 to 4 weeks after bud burst and shows lower caterpillar abundance in evergreen habitats [47]. These differences in habitat and food availability have been identified as a major driver for the phenotypic differences in morphological, life history, ornamental, and behavior traits observed among blue tit populations [50–52].

### Sample collection

Adult blue tits were captured in their nest during the breeding period. After manipulations to measure their morphology, we placed them in a sterilized plastic box for a maximum of 30 s. Out of 522 captures, 165 adults produced a fecal sample in the box. Once we obtained a fecal sample from one or two adults of a nest, we collected fecal samples from their nestlings when they were 8–16 days old. We placed a sterile surface under the nestlings during manipulations to collect fecal samples if a nestling defecated. 179 nestlings out of 742 attempts produced a fecal sample. Once we obtained fecal samples from adults and nestlings, we collected three caterpillars by shaking the trees in a 10 m radius around the nest box. To represent the blue tit diet which varies according to prey availability [53], the first three caterpillars found, regardless of species, were collected. Adult and nestling fecal samples and caterpillars were collected with sterilized tools and stored in 90% ethanol on ice immediately after sampling. Finally, we collected ten oak leaves from the trees where the caterpillars were collected. The leaves were stored in sterile plastic bags on ice immediately after sampling. For each sample type, a negative control was collected in the field as follows. For adults and nestlings, we washed the tools and the box or the surface with 90% ethanol as for actual sample collection and we collected some of the residual ethanol as a sample. For leaves, we opened a sterile plastic bag for the same amount of time as for actual sample collection and sealed. All samples were stored at −20 °C upon returning to the laboratory (0–5 h after sampling).

### DNA extraction

We prepared fecal and caterpillar samples by washing the samples with PBS buffer three times to neutralize the uric acid from the fecal sample and to remove ethanol [54–56]. Leaves sampled around the same nest box were pooled together for the extraction, but caterpillars and feces were extracted individually. The leaves and the pre-wash caterpillar samples were cut into 4–10 mm^2^ pieces. We extracted DNA from the prepared samples using the PowerSoil DNAeasy Extraction Kit (Qiagen, Hilden, Germany) according to the manufacturer protocol.

### DNA library preparation and sequencing

The microbial compositions of the samples were determined by PCR amplification of the V5-V6 regions of the bacterial 16S rRNA gene using chloroplast-excluding 799F/1115R primers [57]. For each sample, the PCR was performed in a 25 μL mixture containing 10 μL of DNA extract, 0.2 μM of each primer, 0.5 U of Phusion Hot Start II High-Fidelity DNA Polymerase (Thermo Fisher Scientific, Waltham, Massachusetts, United States), 1X of Phusion HF Buffer, 0.2 mM of dNTPs, 3% DMSO and following this programme: initial denaturation at 98 °C for 30 s, 35 cycles of denaturation at 98 °C for 15 s, annealing at 64 °C for 30 s and elongation at 72 °C for 30 s and an extension step of 72 °C for 10 min. For PCR negative controls, DNA was replaced by sterile water and for PCR positive controls we used ZymoBIOMICS Microbial Community DNA Standard (Zymo Research, Irvine, California, United States). The sample order was randomized on the PCR plate. All PCR products were visualized on 2% agarose gel and were normalized using SequalPrep Normalization Plate Kit following the manufacturer protocol (Thermo Fisher Scientific). A pool of samples with low-intensity bands on the gels and a pool of samples with high-intensity bands were made for all types of samples (leaves, caterpillars, and fecal samples). The pools were purified with a ratio of 0.7 of AMPure XP using the manufacturer protocol (Beckman Coulter, Brea, California, United States). Quality control of the libraries was assessed as follows: libraries were quantified using the Qubit™ dsDNA HS Assay Kit (Invitrogen, Carlsbad, California, United States) and the NEBNext® Library Quant Kit for Illumina® (New England BioLabs, Ipswich, Massachusetts, United States) and average fragment size was determined using a Bioanalyzer instrument (Agilent Technologies, Santa Clara, California, United States). Before sequencing, the PhiX control library (Illumina, San Diego, California, United States) was spiked into the amplicon pool to improve the unbalanced base composition. Sequencing reaction was performed on an Illumina Miseq at the UQAM CERMO-FC Genomics Platform using the MiSeq reagent kit v3 (2 × 300 cycles; Illumina). The fecal samples were sequenced in three runs and caterpillars and leaves in one run. Sequence data and sample metadata have been deposited in a public data repository (https://figshare.com/projects/Bacterial_microbiota_sequences_from_a_blue_tit_trophic_network/80594).

### Data processing

Illumina sequencing data were processed using DADA2 version 1.12.1 (ref. [58]) to produce an error-corrected table of amplicon sequence variant (ASV) abundances in each sample. We used default parameters for all DADA2 analyses except where specified below. Sequences were trimmed to remove forward and reverse primers and to a length of 201 nucleotides (forward reads) and 264 nucleotides (reverse reads) before ASV identification and merging of paired-end reads to a single ASV sequence. We assigned a taxonomy for all ASVs using the SILVA database [59] and removed all non-bacterial sequences (e.g., chloroplasts, eukaryotes; 1% of sequences) prior to further data processing. We used the decontam R package [60] to remove probable contaminant ASVs that were more abundant in negative controls than in experimental samples. We performed the decontamination on two data subsets: (1) blue tit feces samples and (2) leaf and caterpillar samples (which came from the same sequencing run) and in two steps: (1) using the PCR negative controls and (2) using the extraction and field negative controls as references to identify contaminants. The decontaminated ASV table was rarefied to 4000 reads per sample using the R package phyloseq [61]. We used a rarefaction threshold of 4000 reads to reach a plateau of ASV richness per sample while allowing the inclusion of 70% of all samples. All negative controls were excluded at the rarefaction step because they contained fewer than 4000 sequences. All the positive controls had a similar composition and contained the ASVs expected from the known composition of the mock community. The final dataset contained 37 leaf, 118 caterpillar, and 266 blue tit bacterial microbiota samples from 77 males, 64 females, and 125 nestlings. For details about rarefaction curves and control bacterial taxonomic composition, see the supplementary information (Supplementary Figs. 1 and 2).

### Data analysis

To test for differences in microbiota variance and composition between host types, we performed a multivariate homogeneity test of group dispersions and a permutational multivariate analysis of variance (PERMANOVA) [62] as implemented in the adonis2 function of the vegan R package [63]. To determine if the microbiota composition of leaves and caterpillars or caterpillars and blue tits were more similar than the microbiota composition of leaves and blue tits, we visualized the relative position of the samples from each host type in a PCoA calculated on a chord distance matrix between samples, and we calculated the proportion of ASVs shared between host type bacterial microbiota and visualized it in a Venn diagram.

To understand the spatial structure of each host type microbiota, we analysed leaves, caterpillars, and blue tits separately. For each host type, we quantified variation in bacterial community composition among nest box nested in site nested in forest stand using a nested PERMANOVA on the chord distance matrix between the ASV relative abundance data. To balance the design among nest boxes, we kept only caterpillar samples associated with nest boxes for which a minimum of three caterpillars were available. For blue tit microbiota, we kept only the samples associated with a nest for which adult and nestling fecal samples were available. For details about sample sizes used in all analysis presented in the results, see the supplementary information (Supplementary Table 1). Before all PERMANOVAs, we tested the multivariate homogeneity of group dispersions with the betadisper function of the vegan R package [63] for all combinations of communities and factors.

## Results

### Taxonomic composition of leaf, caterpillar, and bird bacterial microbiota

**Fig. 1:**
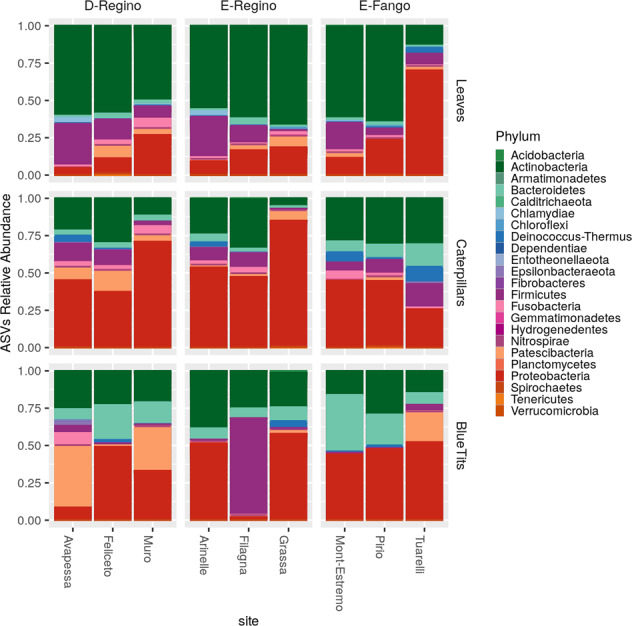
Taxonomic composition at the phylum level for bacterial communities sampled from each host type in each site. Bar height indicates relative abundance of ASVs from different taxa. The sites are grouped by localisation. Avapessa, Feliceto, and Muro are situated in the Regino valley and are dominated by deciduous oak (D-Regino). Arinelle, Filagna, and Grassa are in the Regino valley and are dominated by evergreen oak (E-Regino). Mont-Estremo, Pirio, and Tuarelli are in the Fango valley and are dominated by evergreen oak (E-Fango).

The sequences found in leaves were dominated by *Proteobacteria* (45% of sequences), followed by *Actinobacteria* (26% of sequences), and *Bacteroidetes* (18% of sequences) (Fig. 1). The most abundant bacteria families found in leaves were *Hymenobacteraceae* (16% of sequences), *Burkholderiaceae* (16% of sequences), *Microbacteriaceae* (15% of sequences), *Beijerinckiaceae* (12% of sequences), and *Sphingomonadaceae* (7% of sequences). The ASVs found in caterpillars were dominated by *Proteobacteria* (50% of sequences), *Actinobacteria* (26% of sequences), *Firmicutes* (8% of sequences), and *Bacteroidetes* (6% of sequences) (Fig. 1). The most abundant bacteria families in caterpillars were *Burkholderiaceae* (20% of sequences), *Anaplasmataceae* (10% of sequences), *Corynebacteriaceae* (8% of sequences), *Enterobacteriaceae* (8% of sequences), and *Microbacteriaceae* (5% of sequences). The ASVs found in blue tit feces were dominated by *Actinobacteria* (60% of sequences), *Proteobacteria* (16% of sequences), and *Firmicutes* (14% of sequences) (Fig. 1). The most abundant bacteria families found in Blue tits were *Microbacteriaceae* (8% of sequences), *Pseudonocardiaceae* (8% of sequences), and *Micromonosporaceae* (6% of sequences) (details in Supplementary Table 2 and Supplementary Fig. 3).

### Bacterial microbiota comparisons among host types

**Fig. 2:**
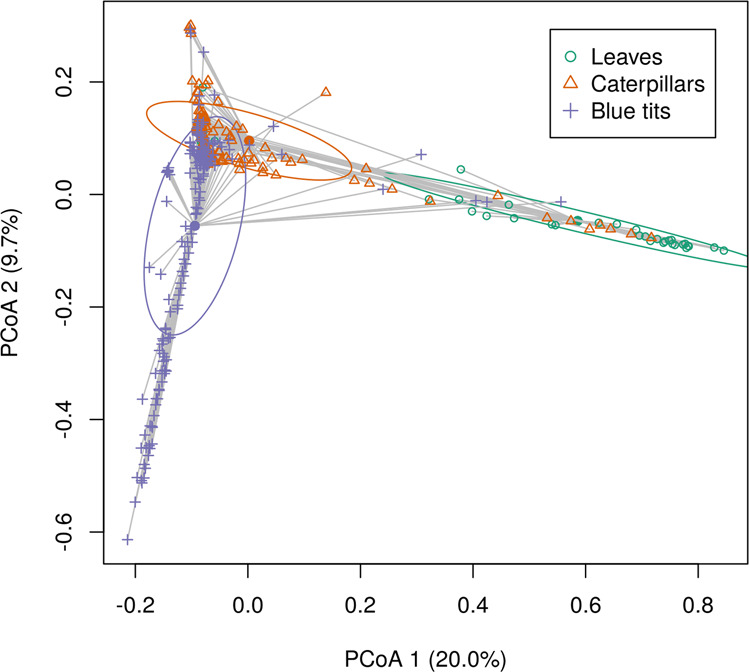
Principal coordinate analysis (PCoA) of microbiota samples from the three host types (leaves, caterpillars, and blue tits). Each sample is connected to the multivariate median of the host type and one standard deviation ellipse (68% confidence ellipses) is presented for each group. PCoA was performed on the chord distance matrix between all samples.

**Fig. 3:**
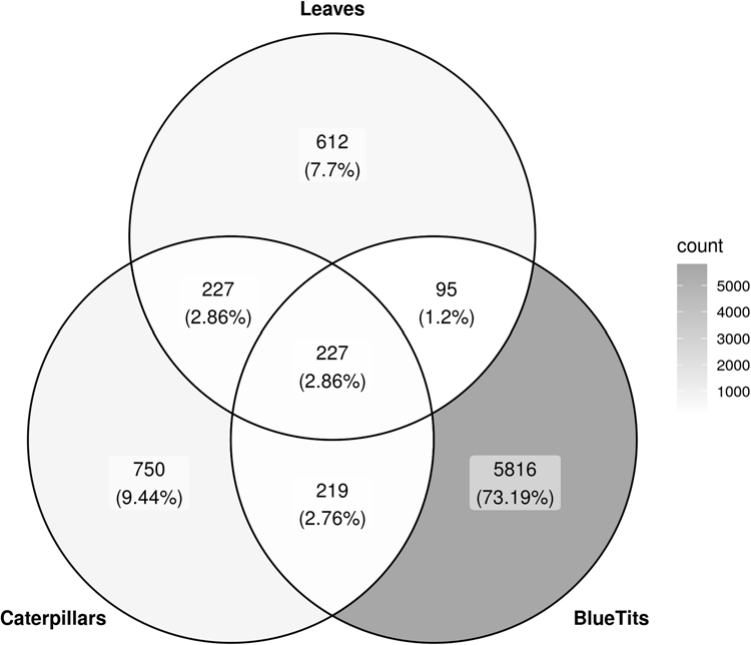
Number of ASVs shared between blue tit, caterpillar and leaf bacterial microbiota. The Venn Diagram shows the number and the proportions in parentheses of ASVs shared between the three host types.

There was a significant difference between bacterial microbiota of leaves, caterpillars, and blue tits (PERMANOVA; Host type: *R*^2^ = 0.044, *P* = 0.001). 227 ASVs, which represent 2.9% of all ASVs found in the samples, were shared among the three host types, but none was shared among all samples. The among-individual variance in bacterial microbiota composition was significantly more important for caterpillars and blue tits than for leaves (Table 1; Multivariate homogeneity test of group dispersions; *P* = 0.001). As predicted, leaf and caterpillar bacterial microbiota as well as caterpillar and blue tit bacterial microbiota were more similar than leaf and blue tit bacterial microbiota. Indeed, in the PCoA (Fig. 2), samples from oak leaves were more similar to those of caterpillars than to those of blue tits, as evidenced by the overlap of confidence ellipses around caterpillar and blue tit bacterial microbiota samples. The Venn diagram (Fig. 3) showed the proportions of ASVs that were shared between host types. Hosts of adjacent trophic levels (leaves-caterpillars and caterpillars-blue tits) shared more common ASVs than leaf and blue tit samples (Fig. 3). Here again, this was consistent with our prediction. Furthermore, the host of higher trophic level (i.e., blue tits) had more unique ASVs than the two other host types. The proportion of ASVs per host type that were shared with the other hosts are presented in Supplementary Figure 4.

**Table 1:** Difference in variance between microbiota samples assessed by a multivariate homogeneity test of group dispersions.

	Difference in variance	*P*
Caterpillars—Leaves	0.218	0.001
Blue tits—Caterpillars	0.003	0.292
Blue tits—Leaves	0.221	0.001

### Bacterial microbiota composition at different spatial scales

**Fig. 4:**
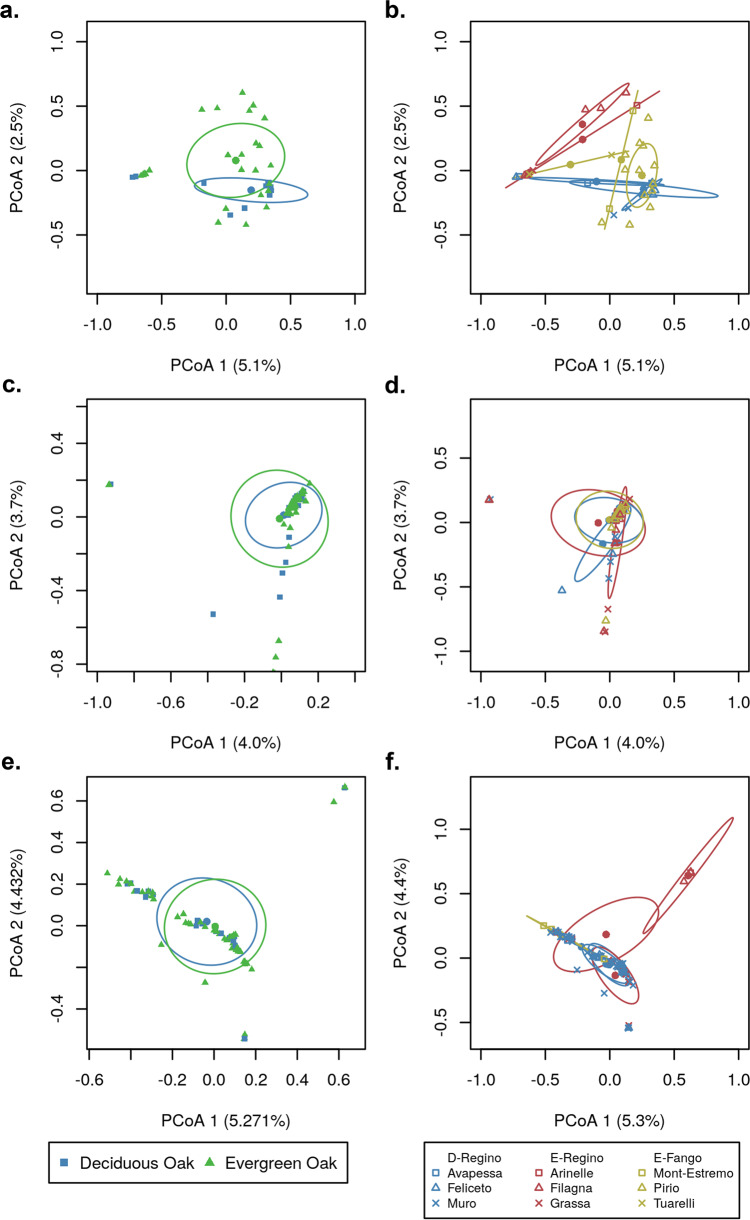
Principal coordinate analysis (PCoA) of microbiota samples from each host type in different locations. Leaf (**a**, **b**), caterpillar (**c**, **d**) and blue tit (**e**, **f**) microbiota sampled in two forest stands (**a**, **c**, **e**) and nine sites (**b**, **d**, **f**) are presented. Avapessa, Feliceto, and Muro are situated in the Regino valley and are dominated by deciduous oak (D-Regino). Arinelle, Filagna, and Grassa are in the Regino valley and are dominated by evergreen oak (E-Regino). Mont-Estremo, Pirio, and Tuarelli are in the Fango valley and are dominated by evergreen oak (E-Fango). A one standard deviation ellipse (68% confidence ellipses) is presented for each group. PCoA were performed on the chord distance matrix between samples for each host type.

The bacterial microbiota structure of each host type varied at the forest stand, the site and the nest box spatial scale (Table 2). Some of the differences in bacterial microbiota composition between forest stands and sites were visible in ordinations of the bacterial microbiota samples (Fig. 4; PCoA). Tree species affected the bacterial microbiota composition of leaves, although the effect of the site was more important than the effect of forest stand (Table 2; PERMANOVA; Forest stand: *R*^2^ = 0.06, *P* = 0.005; Site: *R*^2^ = 0.29, *P* = 0.001). Forest stand and site did not affect the caterpillar bacterial microbiota, but the caterpillars found around a nest box had a more similar bacterial microbiota composition compared with caterpillars around other nest boxes (*R*^2^ = 0.22, *P* = 0.03). The blue tit bacterial microbiota did not vary between the two forest stands, while the site explained a moderate proportion of variation and the nest box explained an important proportion of variation in blue tit bacterial microbiota (Table 2; Forest stand: *R*^2^ = 0.01, *P* = 0.364; Site: *R*^2^ = 0.08, *P* = 0.002; Nest box: *R*^2^ = 0.14, *P* = 0.03). In other words, blue tits had a more similar bacterial microbiota within than among sites and within than among nest boxes. For each host type, all factors passed the multivariate homogeneity test of groups dispersions except the caterpillar bacterial microbiota and the blue tit bacterial microbiota for the factor “site”. Since the effect of site was not significant for caterpillar bacterial microbiota, this was not an issue, but for the site effect on blue tit bacterial microbiota, the effect may be due to differences in variance and not in bacterial composition between samples of different sites.

**Table 2:** Effect of forest stand, site or nest box on the microbiota composition of each host type. The table gives *R*^2^ (P) assessed by nested PERMANOVA (one for each host type).

	Forest stand	Site	Nest box
Leaves	0.06 (0.005)	0.29 (0.001)	N.D.
Caterpillars	0.02 (0.171)	0.08 (0.193)	0.22 (0.03)
Blue tits	0.01 (0.364)	0.08 (0.002)	0.14 (0.03)

## Discussion

### Bacterial microbiota taxonomic composition varies across trophic levels

We described the bacterial microbiota of three host types along a trophic network using 16S rRNA gene amplicon sequencing. The leaf bacterial microbiota of deciduous downy oaks and evergreen holm oaks are dominated by *Proteobacteria*, *Actinobacteria*, and *Bacteroidetes*, as found in other studies of leaf microbiota [64–66]. The phyla *Proteobacteria*, *Actinobacteria*, *Firmicutes*, and *Bacteroidetes* were dominant in caterpillars, like other studies of caterpillar microbiota [34, 67]. The taxonomic composition of blue tit fecal bacterial microbiota was dominated by *Actinobacteria*, *Proteobacteria*, and *Firmicutes*. *Proteobacteria* and *Firmicutes* have been previously reported to dominate bird fecal microbiota [68–70]. Interestingly, we found that *Actinobacteria* were very abundant in bird feces, but this group was not typically dominant in bird fecal microbiota in other studies. Blue tit gut bacterial microbiota composition in our study was similar to that of the closely related great tit (*Parus major*) [71]. In this species the bacterial microbiota was dominated by *Firmicutes*, *Actinobacteria*, and to a lower extent *Proteobacteria* [71]. Blue tits and caterpillars presented a higher among-individual variance in their bacterial microbiota compared to leaves. This was expected for caterpillars that were known to have highly variable microbiota [18, 33, 34] but less expected for blue tits; a previous study of gut microbiota across 59 bird species found that about 20% of the described bacteria were found across all samples [15], although in barn swallow microbiota the most abundant bacteria were present in only half of the samples [72]. Our complementary results on blue tits thus suggested that the level of among-individual variance in bacterial microbiota was variable across bird taxa and populations.

### Evidence of bacterial transfer among hosts

When considering the whole study area, leaf and caterpillar bacterial microbiota as well as caterpillar and blue tit bacterial microbiota were more similar and shared more ASVs in common than leaf and blue tit microbiota, which was consistent with their position in the trophic network (Figs. 2 and 3). Indeed, our results confirm the prediction that hosts of adjacent trophic levels had a more similar bacterial microbiota than hosts separated by two trophic levels. Some bacteria associated with one host type may have been horizontally transferred to another host type in their diet or by direct contact. It is also possible that some environmental variables affected two host types independently resulting in a similar microbiota composition for both. Future experiments will be required to determine the processes that cause this pattern, but our results did indicate clearly that living in spatial proximity led to increased similarity in microbiota composition.

### Variation in bacterial microbiota composition across spatial scales differed among host types

As expected, the leaf bacterial microbiota was different between the two tree species and across the sampled sites (Table 2). Host taxonomy is known as the main factor affecting leaf microbiota composition and the environmental variables associated with location have also been shown to affect bacterial composition [22–25]. One surprising result was that variation in bacterial microbiota composition depended more on the sampling site than on tree species. The tree species in our study system belong to the same genus and it has been shown that closely phylogenetically related host taxa are associated with more similar microbiota [23]. Thus, this may be one of the reasons why in our study, tree taxonomy was not a major driver of bacterial microbiota composition compared to the sampling location.

We found no difference in caterpillar bacterial microbiota composition among forest stands and among sites (Table 2). At a finer scale, the caterpillars sampled around the same nest box had similar bacterial microbiota and in fact nest box location was identified as one of the main determinants of bacterial microbiota diversity (Table 2). Thus, the microenvironment (i.e. the immediate vicinity around a nest box) might be important in shaping the caterpillar bacterial microbiota. However, another study found no similarity in microbiota of *Melitaea cinxia* caterpillars from the same family nest and collected on the same *Plantago lanceolata* host plant individual [73]. Empirical evidence is mixed and more studies looking at the effects of the microenvironment on insect microbiota are needed to resolve the question of how insect microbiota vary across spatial scales.

Our prediction that blue tit microbiota were structured at the three spatial scales was confirmed. Forest stand had a small effect on the bird bacterial microbiota, while site had a moderate effect and the nest box had an important effect on their bacterial microbiota (Table 2). As expected, blue tits from the same nest box (i.e. a breeding pair and their nestlings) had similar gut microbiota. Although we could not separate these causes, our results highlight that the microenvironment was more important than large-scale environment in shaping the blue tit bacterial microbiota. While previous studies have found an effect of the macroenvironment [11, 13, 74] or microenvironment [71, 75] on microbiota, to our knowledge, none has compared the magnitude of these effects.

## Conclusion

This study analysed the composition of the bacterial microbiota of three host types: oak leaves, caterpillars, and blue tits, all part of the same trophic network. Our spatially structured study design provided the opportunity to compare the relations between three host type bacterial microbiota from a trophic network in the wild at different spatial scales. It indicates that, for these three host types, living in spatial proximity led to increased similarity in bacterial microbiota composition. The result suggested a transfer of bacteria from prey to predators in a trophic network composed of a primary producer, a primary consumer, and a secondary consumer. Further studies directly tracking microbial strains across the three trophic levels would be necessary to confirm that a bacterial transfer is occurring in this system. The importance of diet in shaping animal microbiota has been highlighted previously, and our findings add another dimension to our understanding of this relation between host and diet. Our results focused on the taxonomic composition of bacterial microbial communities. Future studies that directly sequence microbial functional genes (e.g., via metagenomic shotgun sequencing) will be required to determine how functions differ among these hosts and to reveal the link between microbial and host functions. Our results also showed that the microenvironment (i.e., the radius of 10 m around the sampled organism) was more important in shaping bacterial microbiota than the large-scale environment (i.e., the site or forest stand sampled) for caterpillars and blue tits. Experimental approaches will be needed to follow up on these results to understand the variables affecting bacterial microbiota at a fine spatiotemporal scale, for example in a nest box.

## Supplementary information

Supplementary Information Description

Supplementary Information

## References

[1] Hooper LV, Bry L, Falk PG, Gordon JI (1998). Host-microbial symbiosis in the mammalian intestine: exploring an internal ecosystem. BioEssays.

[2] Mazmanian SK, Liu CH, Tzianabos AO, Kasper DL (2005). An immunomodulatory molecule of symbiotic bacteria directs maturation of the host immune system. Cell.

[3] Chung H, Pamp SJ, Hill JA, Surana NK, Edelman SM, Troy EB (2012). Gut immune maturation depends on colonization with a host-specific microbiota. Cell.

[4] Heijtz RD, Wang S, Anuar F, Qian Y, Bjorkholm B, Samuelsson A (2011). Normal gut microbiota modulates brain development and behavior. Proc Natl Acad Sci USA..

[5] Erny D, de Angelis ALH, Jaitin D, Wieghofer P, Staszewski O, David E (2015). Host microbiota constantly control maturation and function of microglia in the CNS. Nat Neurosci.

[6] van der Waaij D (1989). The ecology of the human intestine and its consequences for overgrowth by pathogens such as clostridium difficile. Annu Rev Microbiol.

[7] Dinan TG, Stilling RM, Stanton C, Cryan JF (2015). Collective unconscious: how gut microbes shape human behavior. J Psychiatr Res.

[8] Hird SM (2017). Evolutionary biology needs wild microbiomes. Front Microbiol.

[9] Scupham AJ, Patton TG, Bent E, Bayles DO (2008). Comparison of the cecal microbiota of domestic and wild turkeys. Micro Ecol.

[10] Goodrich JK, Davenport ER, Waters JL, Clark AG, Ley RE (2016). Cross-species comparisons of host genetic associations with the microbiome. Science.

[11] Hird SM, Carstens BC, Cardiff SW, Dittmann DL, Brumfield RT (2014). Sampling locality is more detectable than taxonomy or ecology in the gut microbiota of the brood-parasitic brown-headed cowbird (*Molothrus ater*). PeerJ.

[12] Benson AK, Kelly SA, Legge R, Ma F, Low SJ, Kim J (2019). Individuality in gut microbiota composition is a complex polygenic trait shaped by multiple environmental and host genetic factors. Proc Natl Acad Sci.

[13] Musitelli F, Ambrosini R, Rubolini D, Saino N, Franzetti A, Gandolfi I (2018). Cloacal microbiota of barn swallows from Northern Italy. Ethol Ecol Evol.

[14] Muegge BD, Kuczynski J, Knights D, Clemente JC, González A, Fontana L (2011). Diet drives convergence in gut microbiome functions across mammalian phylogeny and within humans. Science.

[15] Hird SM, Sánchez C, Carstens BC, Brumfield RT (2015). Comparative gut microbiota of 59 neotropical bird species. Front Microbiol.

[16] Bili M, Cortesero AM, Mougel C, Gauthier JP, Ermel G, Simon JC (2016). Bacterial community diversity harboured by interacting species. PLoS One.

[17] Sugio A, Dubreuil G, Giron D, Simon J (2015). Plant – insect interactions under bacterial influence: ecological implications and underlying mechanisms. J Exp Bot.

[18] Hannula SE, Zhu F, Heinen R, Bezemer TM (2019). Foliar-feeding insects acquire microbiomes from the soil rather than the host plant. Nat Commun.

[19] White J, Mirleau P, Danchin E, Mulard H, Hatch SA, Heeb P (2010). Sexually transmitted bacteria affect female cloacal assemblages in a wild bird. Ecol Lett.

[20] Schlechter RO, Miebach M, Remus-Emsermann MNP (2019). Driving factors of epiphytic bacterial communities: a review. J Adv Res.

[21] Remus-Emsermann MNP, Lücker S, Müller DB, Potthoff E, Daims H, Vorholt JA (2014). Spatial distribution analyses of natural phyllosphere-colonizing bacteria on *Arabidopsis thaliana* revealed by fluorescence in situ hybridization. Environ Microbiol.

[22] Remus-Emsermann MNP, Tecon R, Kowalchuk GA, Leveau JHJ (2012). Variation in local carrying capacity and the individual fate of bacterial colonizers in the phyllosphere. ISME J.

[23] Rogers TJ, Leppanen C, Brown V, Fordyce JA, LeBude A, Ranney T (2018). Exploring variation in phyllosphere microbial communities across four hemlock species. Ecosphere.

[24] Redford AJ, Bowers RM, Knight R, Linhart Y, Fierer N (2010). The ecology of the phyllosphere: geographic and phylogenetic variability in the distribution of bacteria on tree leaves. Environ Microbiol.

[25] Laforest-Lapointe I, Messier C, Kembel SW (2016). Host species identity, site and time drive temperate tree phyllosphere bacterial community structure. Microbiome.

[26] Kembel SW, Mueller RC (2014). Plant traits and taxonomy drive host associations in tropical phyllosphere fungal communities. Botany.

[27] Appel MH. The chewing herbivore gut lumen: Physicochemical conditions and their impact on plant nutrients, allelochemicals, and insect pathogens. In: Bernays EA (ed.). *Insect-plant interactions*, 1st ed. 1994. CRC Press, Boca Raton, pp 209–23.

[28] Shannon AL, Attwood G, Hopcroft DH, Christeller JT (2001). Characterization of lactic acid bacteria in the larval midgut of the keratinophagous lepidopteran, *Hofmannophila pseudospretella*. Lett Appl Microbiol.

[29] Kukal O, Dawson TE, Kukal O, Dawson TE (1989). Temperature and food quality influences feeding behavior, assimilation efficiency and growth rate of arctic woolly-bear caterpillars. Oecologia.

[30] Vilanova C, Baixeras J, Latorre A, Porcar M (2016). The generalist inside the specialist: gut bacterial communities of two insect species feeding on toxic plants are dominated by *Enterococcus* sp. Front Microbiol.

[31] Priya NG, Ojha A, Kajla MK, Raj A, Rajagopal R (2012). Host plant induced variation in gut bacteria of *Helicoverpa armigera*. PLoS One.

[32] Jones AG, Mason CJ, Felton GW, Hoover K (2019). Host plant and population source drive diversity of microbial gut communities in two polyphagous insects. Sci Rep..

[33] Hammer TJ, Janzen DH, Hallwachs W, Jaffe SP, Fierer N (2017). Caterpillars lack a resident gut microbiome. PNAS.

[34] Whitaker MRL, Salzman S, Sanders JG, Kaltenpoth M, Pierce NE (2016). Microbial communities of lycaenid butterflies do not correlate with larval diet. Front Microbiol.

[35] Stanley D, Geier MS, Hughes RJ, Denman SE, Moore RJ (2013). Highly variable microbiota development in the chicken gastrointestinal tract. PLoS One.

[36] Azcárate-García M, Ruiz-Rodríguez M, Díaz-Lora S, Ruiz-Castellano C, Soler JJ (2019). Experimentally broken faecal sacs affect nest bacterial environment, development and survival of spotless starling nestlings. J Avian Biol.

[37] Devaynes A, Antunes A, Bedford A, Ashton P (2018). Progression in the bacterial load during the breeding season in nest boxes occupied by the Blue Tit and its potential impact on hatching or fledging success. J Ornithol.

[38] Janczyk P, Hall B, Souffrant WB (2009). Microbial community composition of the crop and ceca contents of laying hens fed diets supplemented with *Chlorella vulgaris*. Poult Sci.

[39] Waite DW, Taylor MW (2015). Exploring the avian gut microbiota: current trends and future directions. Front Microbiol.

[40] Pan D, Yu Z (2014). Intestinal microbiome of poultry and its interaction with host and diet. Gut Microbes.

[41] Lewis WB, Moore FR, Wang S (2017). Changes in gut microbiota of migratory passerines during stopover after crossing an ecological barrier. Auk.

[42] Kulkarni S, Heeb P (2007). Social and sexual behaviours aid transmission of bacteria in birds. Behav Process.

[43] Dawkins R (1982). The extended phenotype.

[44] Fisher DN, Haines JA, Boutin S, Dantzer B, Lane JE, Coltman DW (2019). Indirect effects on fitness between individuals that have never met via an extended phenotype. Ecol Lett.

[45] Mennerat A, Perret P, Lambrechts MM (2009). Local individual preferences for nest materials in a passerine bird. PLoS One.

[46] Blondel J, Thomas DW, Charmantier A, Perret P, Bourgault P, Lambrechts MM (2006). A thirty-year study of phenotypic and genetic variation of blue tits in mediterranean habitat mosaics. Bioscience.

[47] Blondel J, Dias PC, Maistre M, Perret P (1993). Habitat heterogeneity and life-history variation of mediterranean blue tits (*Parus caeruleus*). Auk.

[48] Visser ME, Van Noordwijk AJ, Tinbergen JM, Lessells CM (1998). Warmer springs lead to mistimed reproduction in great tits (*Parus major*). Proc R Soc B Biol Sci.

[49] Stenning M. The Blue Tit, 1st ed. (T. & A. D. Poyser, London, UK. 2018) pp 69–109.

[50] Blondel J, Aronson J, Bodiou J-Y, Boeuf G. The mediterranean region: biological diversity in space and time, 2nd ed. 2010. Oxford University Press, Oxford.

[51] Charmantier A, Doutrelant C, Dubuc-messier G, Fargevieille A, Szulkin M (2016). Mediterranean blue tits as a case study of local adaptation. Evol Appl.

[52] Dubuc-Messier G, Réale D, Perret P, Charmantier A (2017). Environmental heterogeneity and population differences in blue tits personality traits. Behav Ecol.

[53] Bańbura J, Blondel J, de Wilde-Lambrechts H, Galan M-J, Maistre M (1994). Nestling diet variation in an insular mediterranean population of blue tits *Parus caeruleus*: effects of years, territories and individuals. Oecologia.

[54] Alda F, Rey I, Doadrio I (2007). An improved method of extracting degraded DNA samples from birds and other species. Ardeola.

[55] Oehm J, Juen A, Nagiller K, Neuhauser S, Traugott M (2011). Molecular scatology: how to improve prey DNA detection success in avian faeces?. Mol Ecol Resour.

[56] Eriksson P, Mourkas E, González-Acuna D, Olsen B, Ellström P (2017). Evaluation and optimization of microbial DNA extraction from fecal samples of wild Antarctic bird species. Infect Ecol Epidemiol.

[57] Chelius MK, Triplett EW (2001). The diversity of archaea and bacteria in association with the roots of *Zea mays* L. Micro Ecol.

[58] Callahan BJ, Mcmurdie PJ, Rosen MJ, Han AW, Johnson AJA, Holmes SP (2016). DADA2: high resolution sample inference from illumina amplicon data. Nat Methods.

[59] Quast C, Pruesse E, Yilmaz P, Gerken J, Schweer T, Yarza P (2013). The SILVA ribosomal RNA gene database project: improved data processing and web-based tools. Nucleic Acids Res.

[60] Davis NM, Proctor D, Holmes SP, Relman DA, Callahan BJ (2017). Simple statistical identification and removal of contaminant sequences in marker-gene and metagenomics data. Microbiome.

[61] McMurdie PJ, Holmes S (2013). phyloseq: an R package for reproducible interactive analysis and graphics of microbiome census data. PLoS One.

[62] Anderson MJ (2001). A new method for non-parametric multivariate analysis of variance. Austral Ecol.

[63] Oksanen J, Blanchet FG, Friendly M, Kindt R, Legendre P, McGlinn D, et al. vegan: Community ecology package. R package version 2.5-7. 2020.

[64] Vorholt JA (2012). Microbial life in the phyllosphere. Nat Rev Microbiol.

[65] Bulgarelli D, Schlaeppi K, Spaepen S, van Themaat EVL, Schulze-Lefert P (2013). Structure and functions of the bacterial microbiota of plants. Annu Rev Plant Biol.

[66] Müller T, Ruppel S (2014). Progress in cultivation-independent phyllosphere microbiology. FEMS Microbiol Ecol.

[67] Chaturvedi S, Rego A, Lucas LK, Gompert Z (2017). Sources of variation in the gut microbial community of *Lycaeides melissa* caterpillars. Sci Rep..

[68] Videvall E, Strandh M, Engelbrecht A, Cloete S, Cornwallis CK (2017). Measuring the gut microbiome in birds: comparison of faecal and cloacal sampling. Mol Ecol Resour.

[69] Lewis WB, Moore FR, Wang S (2016). Characterization of the gut microbiota of migratory passerines during stopover along the northern coast of the Gulf of Mexico. J Avian Biol.

[70] Sun CH, Liu H-Y, Zhang Y, Lu C-H (2019). Comparative analysis of the gut microbiota of hornbill and toucan in captivity. Microbiologyopen.

[71] Teyssier A, Lens L, Matthysen E, White J (2018). Dynamics of gut microbiota diversity during the early development of an avian host: evidence from a cross-foster experiment. Front Microbiol.

[72] Ambrosini R, Corti M, Franzetti A, Caprioli M, Rubolini D, Motta VM (2019). Cloacal microbiomes and ecology of individual barn swallows. FEMS Microbiol Ecol.

[73] Minard G, Tikhonov G, Ovaskainen O, Saastamoinen M (2019). The microbiome of the *Melitaea cinxia* butterfly shows marked variation but is only little explained by the traits of the butterfly or its host plant. Environ Microbiol.

[74] Godoy-Vitorino F, Leal SJ, Díaz WA, Rosales J, Goldfarb KC, García-Amado MA (2012). Differences in crop bacterial community structure between hoatzins from different geographical locations. Res Microbiol.

[75] Lucas FS, Heeb P (2005). Environmental factors shape cloacal bacterial assemblages in great tit *Parus major* and blue tit *P. caeruleus* nestlings. J Avian Biol.

