# Supplementary material for: Bacterial microbiota similarity between predators and prey in a blue tit trophic network
Source: ISME J. 2021 Feb 12;15(4):1098–107. doi: 10.1038/s41396-020-00836-3 (PMC8115664; doi:10.1038/s41396-020-00836-3)
Supplement: Supplementary file 1 — Supplementary Information Description [file 41396_2020_836_MOESM1_ESM.pdf]

Supplementary Information Description

The Supplementary Information is a PDF file containing rarefaction curves for microbiota samples (Figure 1), sample sizes for every analysis in the manuscript (Table 1), taxonomic composition of controls (Figure 2) and of samples (Table 2 and Figure 3), and the proportion of ASVs shared between host types (Figure 4).
