# Supplementary material for: Bacterial microbiota similarity between predators and prey in a blue tit trophic network
Source: ISME J. 2021 Feb 12;15(4):1098–107. doi: 10.1038/s41396-020-00836-3 (PMC8115664; doi:10.1038/s41396-020-00836-3)
Supplement: Supplementary file 2 — Supplementary Information [file 41396_2020_836_MOESM2_ESM.pdf]

a.

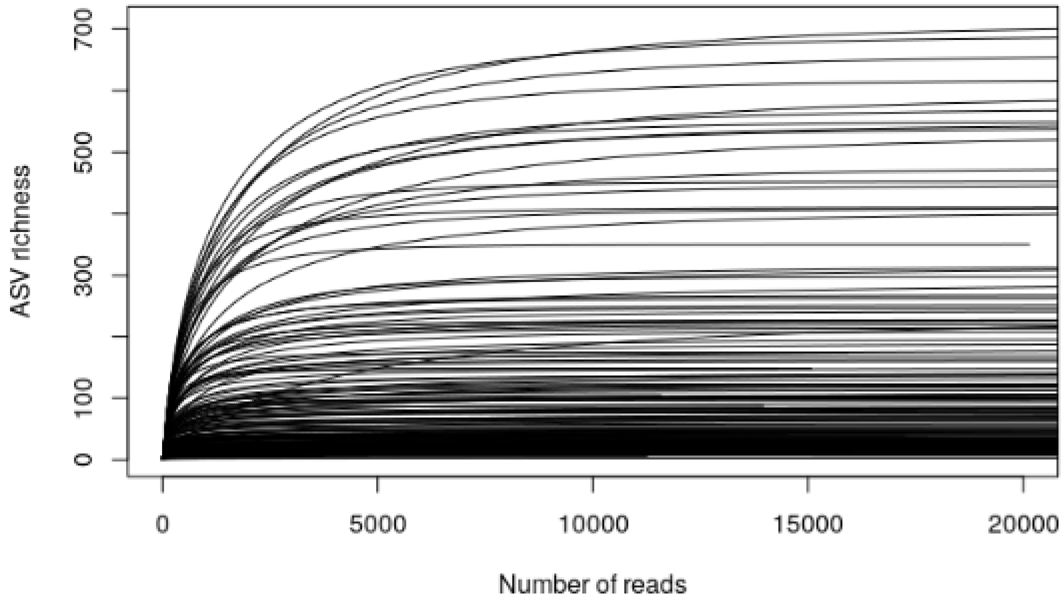

b.

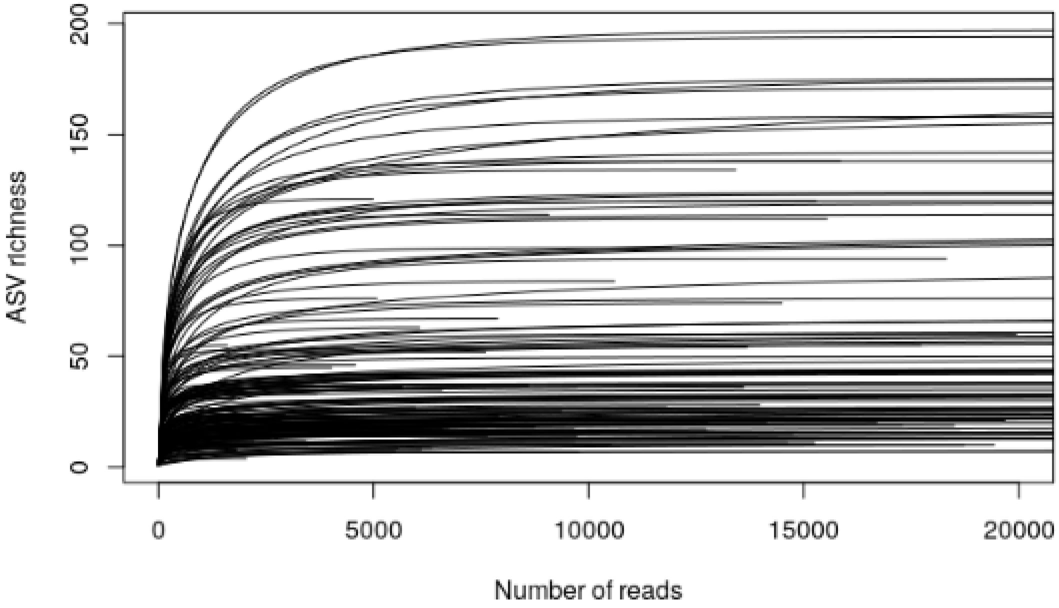

Supplementary Figure 1. Rarefaction curve for a. blue tit microbiota samples and b. leaves and caterpillar samples. The rarefaction threshold was fixed to 4000 reads per sample, which was sufficient to reach a plateau in ASVs richness for all samples.

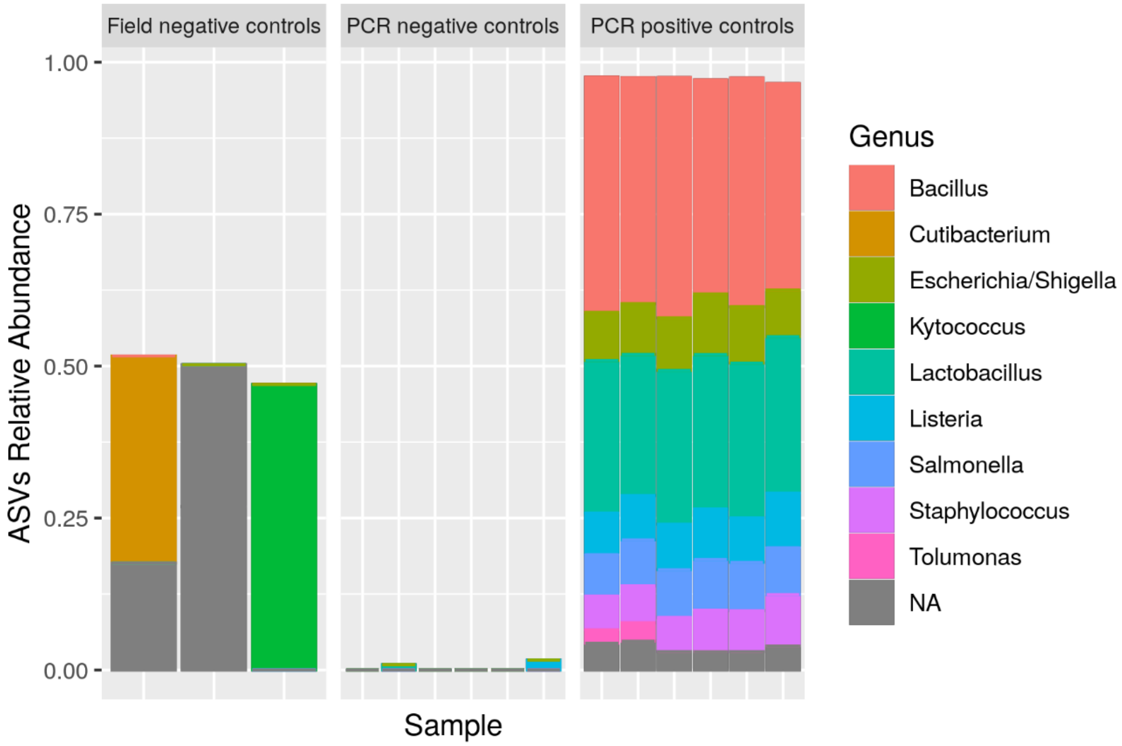

Supplementary Figure 2. Taxonomic composition at the genus level of the 30 most abundant ASVs in PCR positive controls from a mock community, field negative controls and PCR negative controls. Bar height indicates relative abundance of ASVs from different taxa. Only three of the genera (*Escherichia*/*Shigella*, *Lactobacillus*, and *Staphylococcus*) abundant in the control samples were also among the 30 most abundant ASVs found in leaf, caterpillar or blue tit microbiota samples.

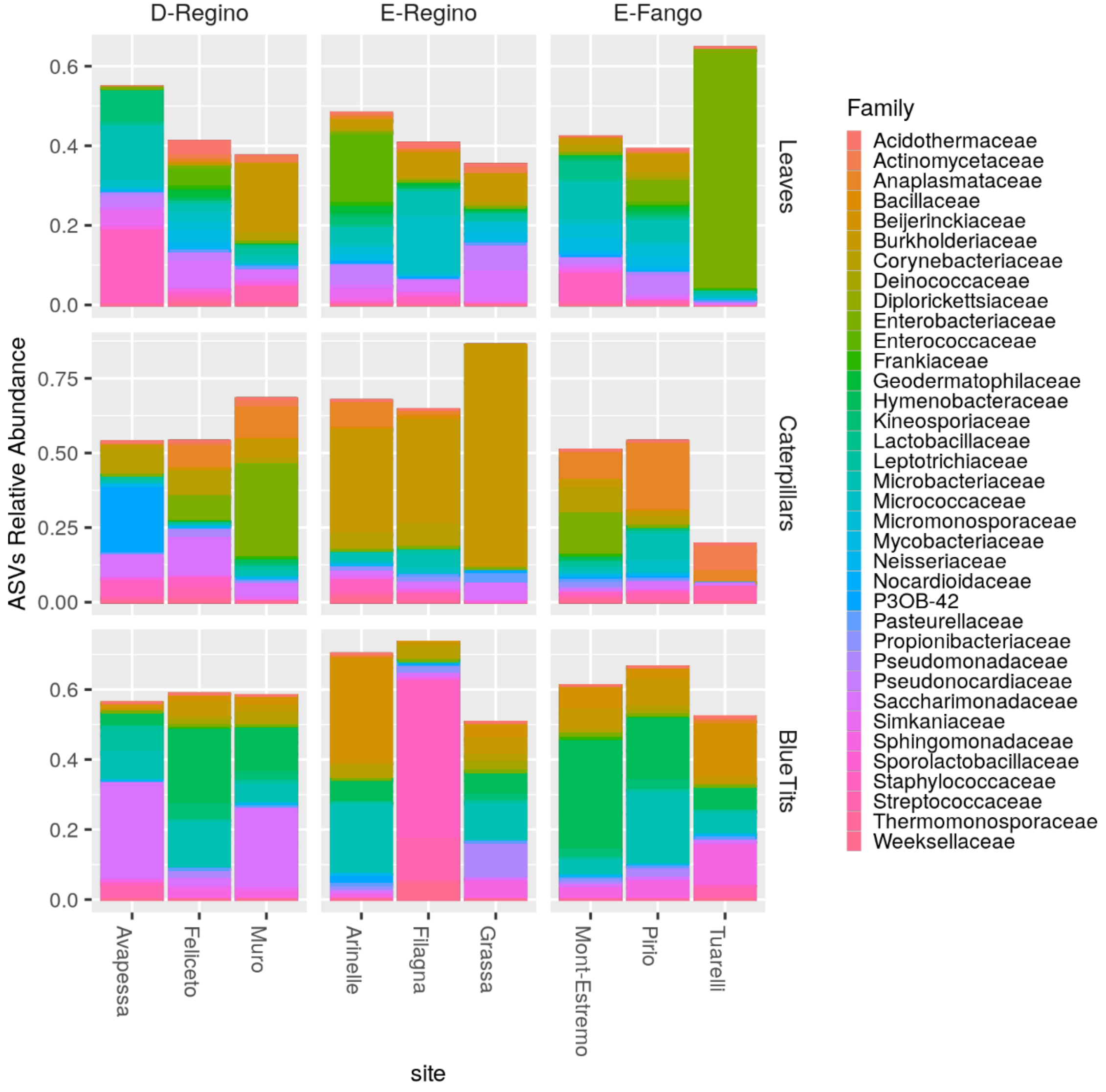

Supplementary Figure 3. Taxonomic composition at the family level of the 100th most abundant ASVs in all samples for each host type in each site. The sites are grouped by localisation. Avapessa, Feliceto, and Muro are situated in the Regino valley and are dominated by deciduous oak (D-Regino). Arinelle, Filagna, and Grassa are in the Regino valley and are dominated by evergreen oak (E-Regino). Mont-Estremo, Pirio, and Tuarelli are in the Fango valley and are domінted by evergreen oak (E-Fango).

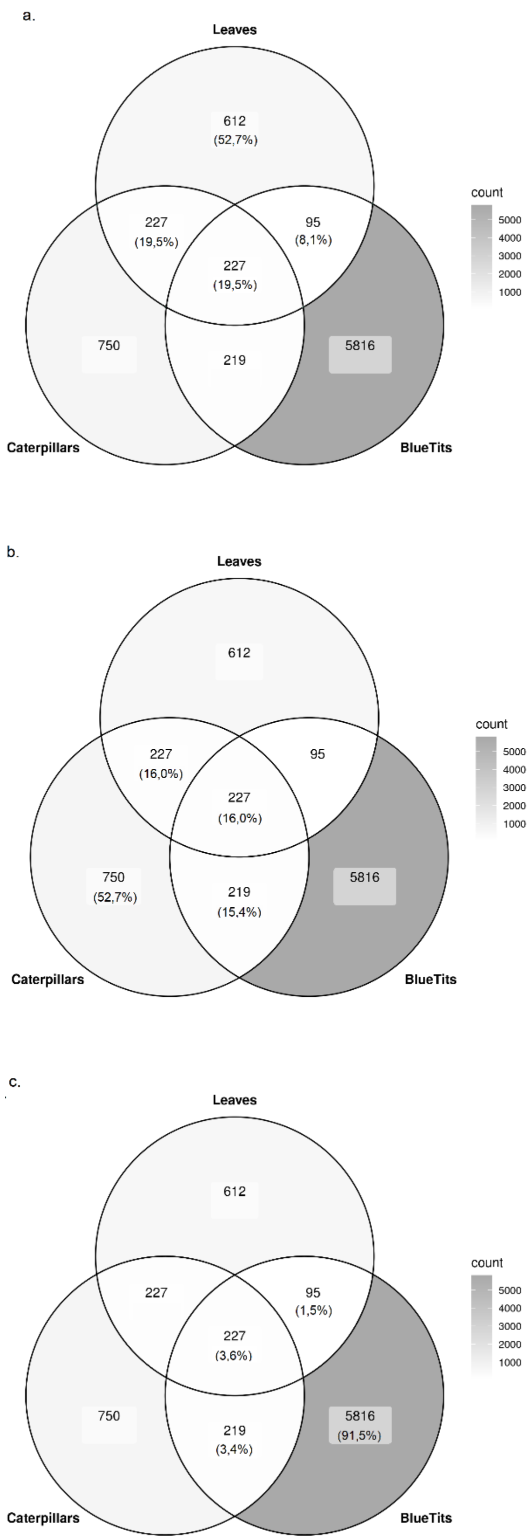

Supplementary Figure 4. Proportion of ASVs from a. leaf, b. caterpillar, and c. blue tit bacterial microbiota samples shared or not with other host types.

Supplementary Table 1. Sample size of all analysis for each host type and site. Avapessa, Feliceto, and Muro are situated in the Regino valley and are dominated by deciduous oak (D-Regino). Arinelle, Filagna, and Grassa are in the Regino valley and are dominated by evergreen oak (E-Regino). Mont-Estremo, Pirio, and Tuarelli are in the Fango valley and are dominted by evergreen oak (E-Fango). The tables and figures associated with each sample sizes are also provided.

		**D-Regino**			**E-Regino**			**E-Fango**		
		**Avapessa**	**Feliceto**	**Muro**	**Arinelle**	**Filagna**	**Grassa**	**Mont-Estremo**	**Pirio**	**Tuarelli**
Host type comparison (Fig. 1, 2, 3; Table 1)	Leaves	2	3	4	5	2	2	4	13	2
	Caterpillars	9	14	10	19	9	4	21	30	2
	Blue tits	11	20	11	29	12	6	37	70	4
Spatial structure (Fig. 4; Table 2)	Leaves	3	4	4	2	5	2	2	13	2
	Caterpillars	9	4	14	6	14	7	0	15	0
	Blue tits	5	13	22	0	6	6	3	53	0

Supplementary Table 2. Relative abundance (% of sequences) of phyla found in each host type. The most abundant phyla discussed in the article text are indicated in bold.

	**Leaves**	**Caterpillars**	**BlueTits**
Acidobacteria	<0.01	<0.01	<0.01
Actinobacteria	**0,26**	**0,26**	**0,60**
Armatimonadetes	<0.01	<0.01	<0.01
Bacteroidetes	**0,18**	**0,06**	0,02
Calditrichaeota	<0.01	<0.01	<0.01
Chlamydiae	<0.01	<0.01	0,01
Chloroflexi	<0.01	<0.01	<0.01
Deinococcus-Thermus	0,02	0,03	<0.01
Dependentiae	<0.01	<0.01	<0.01
Entotheonellaeota	<0.01	<0.01	<0.01
Epsilonbacteraeota	<0.01	<0.01	<0.01
Fibrobacteres	<0.01	<0.01	<0.01
Firmicutes	0,04	**0,08**	**0,15**
Fusobacteria	<0.01	0,03	0,02
Gemmatimonadetes	<0.01	<0.01	<0.01
Hydrogenedentes	<0.01	<0.01	<0.01
Nitrospirae	<0.01	<0.01	<0.01
Patescibacteria	0,04	0,04	0,03
Planctomycetes	<0.01	<0.01	<0.01
Proteobacteria	**0,45**	**0,50**	**0,16**
Spirochaetes	<0.01	<0.01	<0.01
Tenericutes	<0.01	<0.01	<0.01
Verrucomicrobia	<0.01	<0.01	<0.01
